# Review on Autoimmune Reactions in Female Infertility: Antibodies to Follicle Stimulating Hormone

**DOI:** 10.1155/2012/762541

**Published:** 2011-10-05

**Authors:** Kadri Haller-Kikkatalo, Andres Salumets, Raivo Uibo

**Affiliations:** ^1^Department of Immunology, Institute of General and Molecular Pathology, University of Tartu, Ravila Street 19, Biomedicum, Tartu 50411, Estonia; ^2^Department of Obstetrics and Gynecology, University of Tartu, L. Puusepa 8, 51014 Tartu, Estonia; ^3^Competence Centre on Reproductive Medicine and Biology, Tiigi 61b, Tartu 50410, Estonia

## Abstract

Female fertility can be affected by diseases or dysfunctions of reproductive tract, neuroendocrine system, and immune system. Reproductive autoimmune failure can be associated with overall activation of immune system or with immune system reactions specifically directed against ovarian antigens. Majority of the antiovarian autoantibodies are directed against **β**-subunit of follicle stimulating hormone (anti-FSH). This paper summarizes a current clinical classification of female infertility in the context of general activation of autoimmunity and antiovarian autoimmunity by describing serum anti-FSH. The presence of naturally occurring anti-FSH in healthy women will be discussed. In addition, the putative impairment of ovarian folliculogenesis in case of increased production of those antibodies in infertile women will be characterized.

## 1. Introduction

Infertility is a condition that affects a couple and is defined as the lack of conception after an arbitrary period of 12 months without using any contraception [1]. These couples comprise the infertile and the sterile members of the population, for whom is no possibility of natural pregnancy, and the remainder who are subfertile [2]. The latter inadvertently includes normal fertile females who failed to conceive by chance during the 12 or 13 opportunities a woman has per year [1]. Infertility contributes a great proportion to overall reproductive ill health, since there are ~60–80 million infertile couples (~15% of couples) around the world [2]. Minor fertility impairment is seen in both partners more frequently than expected (>70% of infertile couples) [2]. Although infertility *per se *may not threaten physical health, it may have a serious impact on the mental and social well-being of couples and may result in detrimental social consequences, such as divorce or ostracism [2]. In addition, infertility contributes to low birthrate, which is a major social and national issue in developed countries.

Infertility represents an increasing medical problem. A progressive decrease in fertility rate has been indicated since 1955 [1]. The decrease is associated with both medical and nonmedical factors. Women's age is the major determinant of the average time required to conceive. The highest live birth rates are in the age group of 25–30 years and declines sharply after the age of 35 [3]. Also, the duration of infertility contributes meaningful information to the estimation of future fertility [4]. Chromosomal aberrations, monogenic diseases, endocrine dysfunctions, sexually transmitted diseases (STDs), and immune system dysfunctions are medical situations, which can contribute both to male and female infertility. Unfortunately, still in about 10%–20% of couples, the infertility cause remains unknown [2]. However, autoimmune mechanisms may be the case in those couples and have been associated with premature ovarian failure (POF), “subclinical” ovarian failure and with recurrent pregnancy loss [1].

Nowadays, when the utilization of assisted reproduction technologies has improved the prospects of infertility treatment, still every second infertile couple seeks for medical advice [2]. First child following *in vitro* fertilization (IVF) was born in 1978 [5]. Today, approximately 2.5% of newborns account for IVF-treated couples in European countries, which remains somewhat lower when compared to Nordic countries [6]. Regardless of constant improvement of pregnancy rate in IVF, the success rates are still around 30% per cycle [6]. Autoimmunity and the presence of autoantibodies have been invoked as a possible mechanism of IVF failure. There are contradicting data regarding the importance of certain antibodies to damage directly the preimplantation embryo, interfering with implantation process or formation of placenta [7–11]. Consequently, the overall activation of the immune system in female infertility has been suggested [12]. 

For the purpose of improving infertility treatment, the mechanisms of immune system associated with natural reproduction as well as with infertility should be carefully evaluated. This paper summarizes a current clinical classification of female infertility in the context of general activation of autoimmune processes and antiovarian autoimmunity by describing serum antibodies to follicle stimulating hormone (FSH).

## 2. Autoimmunity

Active tolerance mechanisms are required to prevent inflammatory responses to the many innocuous air-borne and food antigens that are encountered at mucosal surfaces. However, the most important aspect of tolerance is self-tolerance, which prevents the body from mounting an immune attack against its own tissues—prevention from autoimmune reactions. Autoimmunity is associated with a dysbalance of various components of the immune response and with the development of autoantibodies directed against normal host antigens. The susceptibility to autoimmune reactions is regulated at several levels [13]. The proliferation of mature T-lymphocytes in response to either self- or foreign antigenic stimuli is affected by the nature and strength of antigenic peptide-MHC (major histocompatibility complex) stimulation [13, 14]. Human leukocyte antigen (HLA)-class II molecules influence the stability of the antigenic-peptide-HLA complex in an allele-specific manner, affecting the induction of central tolerance [13]. As revealed by the studies on anti-insulin autoimmunity, the stimulation provided by antigenic peptide-MHC stimulation could also be modulated by genetic variations of the insulin gene, influencing the gene expression in the thymus [15, 16]. Tissue-specific autoimmunity appears to be additionally dependent on local factors, including infection-related tissue damage [13], iatrogenic manipulations [17], and the level of autoantigen in periphery [18, 19]. Thus, the expansion of cells responding to low-affinity ligands (self-antigen) or anomalies in the deletion of high-affinity autoreactive T-cells can lead to autoimmune reactions [14]. Once an autoimmune disease has been developed, a wider range of autoimmune reactions may progress, meaning that an individual may develop more than one autoimmune disease [20].

## 3. Reproductive Autoimmune Failure in Women

Female fertility is regulated by a series of highly coordinated and synchronized interactions in the hypothalamic-pituitary-ovarian axis. Therefore, female fertility can be affected by diseases or dysfunctions of reproductive tract, neuroendocrine system, and immune system or by any severe or exhausting general disease. The etiology of female infertility in a diagnostic and treatment point of view is summarized in Table 1 (based on the guidelines provided by [1, 2]). The reproductive autoimmune failure syndrome was originally described by Gleicher et al. in women with endometriosis, infertility and increased autoantibodies [21]. Autoimmune mechanisms as well as an increased production of multiple autoantibodies are involved in such infertility disorders as POF, endometriosis, polycystic ovary syndrome (PCOS), unexplained infertility, and repeatedly unsuccessful IVF attempts and may be responsible for the pathophysiology of preeclampsia or spontaneous abortions, as stated in many original articles as well as discussed in reviews (Table 2) [19, 22–25]. Although not many studies have been performed on humans, the role of cellular immunity in ovarian autoimmunity, in addition to humoral immunity, has been detected both locally in the ovary [26] as well as in periphery [27]. However, due to the technical difficulties in everyday laboratory work, most clinical studies are restricted to detecting serum antibodies in order to define autoimmune activation in a patient. 

In Western Europe and North America, where tubal diseases are relatively uncommon, endocrine dysfunctions can be identified in about 10%–20% of women presenting with infertility [28]. Most common cause for hypergonadotropic hypogonadism is POF [1]. POF is defined as secondary amenorrhea with elevated gonadotrophin levels observed under the age of 40 and affect 1%-2% of women of the general population [1]. POF is highly heterogeneous condition and can be associated with autoimmune disorders, ovarian surgery, iatrogenic causes such as chemoradiotherapy, systemic diseases such as galactosaemia, or with genetic factors [1]. In more than half of the cases, the development of POF has been associated with autoimmune reactions to ovarian tissue [29, 30]. An investigation of antiovarian autoimmune reactions and autoantibodies may be severely hampered by the fact that POF represents an end-stage of disease. By the time when the disease in a women is diagnosed, she has exhausted her follicular supply and, presumably, also the target antigen for the autoimmune attack on her ovary. Thus, the autoimmunity causal of POF can be difficult to detect retrospectively. Regardless of that, high prevalence of antiovarian antibodies (AOA) (30%–67%) and others organ- and nonorgan-specific autoantibodies have been observed in patients with POF [29, 31, 32].

Normogonadotropic anovulation represents about 50% of women with an endocrine cause of infertility and includes mostly the patients with PCOS. PCOS affects up to 4%–10% of all women of reproductive age [33, 34]. PCOS is characterized by polycystic ovaries, oligoanovulation, insulin resistance, and hyperandrogenism or hyperandrogenaemia [35, 36]. Infertility in PCOS is associated with an alteration in folliculogenesis and in the selection of the dominant follicle leading to anovulation [37]. An autoimmune mechanism has also been suggested in some cases of PCOS, where increased prevalence of AOA and common organ- and nonorgan-specific autoantibodies has been detected [19, 22, 25].

Tubal factor infertility accounts for 10%–30% in developed countries and up to 85% in developing countries of reported cases of infertility [38]. Decreased fecundity may be attributed to impaired ovum transport due to fimbrial damage and/or adnexal adhesions. The factors responsible for tubal disease are diverse and include infections, pelvic surgery, and endometriosis. Pelvic inflammatory disease (PID) represents the link between STD and infertility. In majority of cases, acute PID results from acute bacterial endometritis and salpingitis. Most of the long-term consequences of PID, however, stem from the destruction of normal tubal structure, with or without tubal occlusion [1]. While in developed countries, there has been a decline in the incidence of STD salpingitis and correspondingly in PID by the end of 1980s, a significant rise of STD in Eastern Europe and central Asia, has been documented at the beginning of 1990s [2, 39, 40]. The incidence of infertility following the acute PID depends on various factors and varies from 6% to 60% [1]. In addition, there is a silent, relatively asymptomatic PID, which could be the case in up to 80% of chlamydial infections [41]. Genital infection of *Chlamydia trachomatis *is currently the most common bacterial STD (in 20%–40% of cases) and it coexists with the infection of *Neisseria gonorrheae *in 25%–50% of cases [1]. Manifestation of tubal destruction, however, is dependent also from the ability to activate autoimmune inflammation. During chlamydial infection, similar to most infections, the synthesis of heat shock proteins (HSPs) is strongly upregulated. HSPs are the major antigens and can induce a strong immune response [42]. Because there is a strong amino acid sequence homology between microbial and human HSPs, the induced immune response against microbial HSPs may incite an autoimmune inflammatory reaction in the host, culminating in tubal damage [42, 43]. 

Endometriosis is characterized by the growth of endometrial tissue outside the uterine cavity. It is a common disorder, affecting 10%–20% of all women of reproductive age [44, 45]. The most frequent clinical presentations of endometriosis include dysmenorrhea, pelvic pain, dyspareunia, infertility, and pelvic mass. In addition to distorted pelvic anatomy, altered peritoneal function, impaired implantation, and endocrine and ovulatory abnormalities, the alterations in humoral and cell-mediated immune system reactions contribute to the endometriosis-associated female infertility [46]. Moreover, endometriosis has been labelled an “autoimmune syndrome”. Classical autoimmune diseases, as well as endometriosis, are characterized by polyclonal B-cell activation and production of multiple different autoantibodies [21]. About 40%–60% of patients with endometriosis have elevated autoantibody titers when tested against a panel of autoantigens [47]. They often possess specific antiendometrial antibodies [43, 48, 49], but also AOA, antinuclear autoantibodies (ANA), smooth muscle autoantibodies (SMA), and antiphospholipid antibodies (APA) [23, 50, 51].

Approximately 10%–20% of couples who are unable to conceive are determined to have unexplained infertility [2]. Unexplained infertility is a term applied to an infertile couple whose standard investigations (semen analysis, tubal patency, and laboratory assessment of ovulation) yield normal results. A longer period has been suggested to be required for this group of patients to achieve pregnancy without treatment, as 70% of fertility rate is achieved in two years for the group of unexplained infertility, whereas only nine months are required for the fertile group to achieve the same rate [52]. However, about 20%–30% of these patients remain infertile even after 9 years of attempting to conceive [53]. Therefore, unexplained infertility appears to represent either the lower extreme of normal distribution of fertility, or it arises from a defect in fecundity that cannot be detected by the routine infertility evaluation [2, 54–56]. Dysregulation in immune system reactions with enhanced production of autoantibodies is putative etiologic candidate for this group of patients [32, 57, 58]. 

Thyroid autoantibodies have been associated with recurrent pregnancy loss, POF, and repeatedly unsuccessful IVF attempts [11, 59, 60]. This is hypothetically explained by the fact that organ-specific autoimmune diseases, like thyroiditis, may develop secondary to some basic cellular abnormality that directly affects pregnancy outcome [60, 61]. Repeated IVF failure has been associated with increased prevalence of many autoantibodies, including AOA, APA, ANA, SMA, and antisperm antibodies [61, 62]. Therefore, the failure in differentiation of uterine T-cells into T-regulatory cells, as a key determinant of fertility in women has been suggested to be a case in unexplained infertility [58]. Since the prevalence of AOA in unexplained infertility and POF has been detected similar, the unexplained infertility was suggested to represent an early stage of autoimmune POF [32]. 

The impact of a particular autoantibody on the pathogenesis of infertility is not uniformly understood. ANA could interfere with early implantation of embryo and SMA could alter the fallopian tube function [23]. It is concluded that APA may be involved in uterine vascular modifications affecting implantation processes [63]. Except AOA in ovulatory dysfunctions and disease-specific autoantibodies described in case of endometriosis [43, 48, 49, 64], autoantibodies detected in infertile patients are usually not specific to infertility or to the gynaecological diseases leading to infertility. Furthermore, the number of detectible autoantibodies, in particular, has been proposed to predict the pregnancy rate of IVF treatment [65]. Therefore, some studies suggest lesser importance of specific autoantibodies and stress the key role of overall activation of the immune system in reduced fecundity [12, 65]. Consequently, the autoimmune-associated infertility might be a polyclonal event characterized by immunological defects at the T-cell level which, similarly to classical autoimmune diseases, may manifest itself in abnormal antibody production [66]. 

### 3.1. Antiovarian Autoantibodies

Although the presence of AOA immunoglobulin G (IgG) has been documented in different groups of infertile patients (Table 2), there are no epidemiological studies of ovarian autoimmunity. Using an estimated prevalence of autoimmune POF, about 1.1 million women potentially have ovarian autoimmunity in US, which makes ovarian autoimmunity far more common than Addison's disease, myasthenia gravis, or systemic lupus erythematosus [67]. 

Some antibodies in the pool of AOA are suggested to associate with a direct action on ovarian tissue, whereas others have no such effects, similar to autoantibodies in other autoimmune diseases [67]. Therefore, it is possible, that several different antigens are involved in ovarian autoimmunity, as both ovarian cellular and zona pellucida/oocyte antibodies have been reported. Antioocyte antibodies were identified already in 1966, and this was also one of the first descriptions of antiovarian autoimmunity [76]. High prevalence of antizona pellucida antibodies have been detected in infertile women, but also in healthy fertile women and even in men [22]. Antibodies to steroid cells (SCA) are more prevalent in POF patients with Addison's disease (73%–87%), but rare in those patients with other autoimmune disease (0%–8%) or in 0%–10% of patients with isolated POF [22]. Steroidogenic enzymes such as 17*α*-hydroxylase, desmolase (P450-side chain cleavage), 3*β*-hydroxysteroid dehydrogenase, and 21-hydroxylase have been detected as the molecular targets of SCA [69, 77–80]. The aldehyde dehydrogenase and selenium-binding protein 1 [81], human heat-shock protein 90-beta [82] and antialpha-enolase [83] has recently been identified as unique antigens in antiovarian autoimmunity associated with POF and infertility. Gonadotrophin receptors have been also investigated as a potential autoantibody targets. While antibodies against LH receptor were first identified in 30% of IVF patients and in 50% of infertile patients with endometriosis [68, 84], only few cases of POF patients possessing antibodies to FSH receptor was documented [85]. A later study on FSH receptor blocking ability of these antibodies has allowed questioning the pathophysiological role of anti-FSH receptor antibodies in ovarian failure [70]. 

Although blocking antibodies are usually considered to interact with receptors, the FSH and LH activity-inhibiting antibodies could also directly recognize gonadotrophins themselves. The presence of anti-FSH and anti-LH antibodies in poor responder IVF patients has been associated with immunization against exogenous gonadotrophins [72]. Until recently, antigonadotrophin antibodies had been described only in POF patients and that with conflicting results. By using different antibody assays, some authors suggest the importance of only anti-LH antibodies [30], while others evidence the association of POF with anti-FSH antibodies [71]. The latter group presented antibodies against *β*-subunit of FSH in nearly all of the studied AOA-positive POF patients and no anti-LH activity was detected in these samples. Moreover, these antibodies recognized epitopes all over the *β*-subunit molecule, but a region between amino acids 78 and 93 (V14D) was predominantly recognized in all samples, probably representing the immunodominant epitope [71]. The antibodies detected could readily explain the ovarian failure in POF patients, since this part of the *β*-subunit of FSH molecule is directly involved in determining the specificity of receptor binding [86]. The ability of anti-FSH to inhibit the function of FSH hormone has been detected in men [87]. We have looked for the information regarding to the presence of anti-FSH IgG, but also IgA and IgM, in different etiologic groups of female infertility, in healthy women and during pregnancy. Pregnancy itself is accompanied with a suppression of the development of new ovulating follicles. This ovulatory quiescence is due to an inhibition of the pituitary during pregnancy, as seen in the decreased response of FSH and LH to GnRH administration [88]. In addition, we have been interested in the etiologic factors for overproducing anti-FSH antibodies of all subtypes in infertile women as well as the putative pathological role of these antibodies on folliculogenesis or on effectiveness of infertility treatment.

## 4. Follicle Stimulating Hormone

### 4.1. Regulation of Gonadal Function by FSH

FSH is one of the two pituitary gonadotrophins involved in the regulation of the gonadal function. In females, FSH targets the receptor expressed only on granulosa cells and induces the maturation of ovarian follicle [89]. FSH can influence the development of preantral follicles via paracrine factors [90]. However, growth of antral follicles becomes critically dependent on FSH support, making a preovulatory follicle capable of ovulation and forming a corpus luteum in response to the mid-cycle surge of LH [91]. The role of FSH and its signalling system is central in the normal reproductive function since mutations in human FSH and its receptor are associated with altered ovarian responses to the hormone, resulting in various degrees of reduced reproductive function [92, 93].

### 4.2. Coding Genes and Molecular Structure of FSH

FSH is a heterodimer, consisting of an *α*-subunit common to all gonadotrophins (92 amino acids) and a unique *β*-subunit (111 amino acids in FSH). Glycosylation of the gonadotrophins is important in circulatory persistence, clearance and in bioactivity [94]. In a solvent environment, two FSH molecules form an asymmetric unit in clasped hands-like fashion [86]. The *α*-subunit carboxy-terminus as well as carbohydrate residues linked to the *α*-subunit have been implicated in receptor binding and activation [86, 94]. However, there is a cysteine noose, or determinant loop on the *β*-subunit of FSH molecule (between amino acids 87 and 94), the residues of which (Asp 88, Asp 90, and Asp 93) play a role in determining the specificity of FSH receptor binding [86].

The receptor-binding and hormone specificity determining *β*-subunit of FSH hormone is coded by *FSHB *gene at the 11p13 [86]. Haplotype analysis has revealed two most prevalent variants of *FSHB* gene—HAP1 and HAP13 [95]. These two core haplotypes have been suggested to be associated with female's fecundity [95], but the association with autoimmunity to FSH through gene expression in central tolerance induction towards FSH had not been studied.

## 5. Anti-FSH Antibodies Being Primarily Natural Antibodies

We observed the physiological presence of antibodies directed to FSH in a control group of healthy nonpregnant women, significantly lower values of IgG and IgM but not IgA anti-FSH antibodies during uncomplicated pregnancy [75], and increased levels of these antibodies in infertile women [73, 75]. A total of 233 consecutive women undergoing IVF treatment in Estonia constructed the infertility patient group in our studies. We have demonstrated the production of anti-FSH IgM antibodies associated with peripheral FSH hormone levels. This association was detected among patients with tubal and male factor infertility [73]. The production of autoantibodies can be enhanced if there is elevated level of autoantigen, as elevated FSH levels and AOA in case of premature menopause [19]. Similarly, autoantibodies and insulin levels in pancreatic *β* cells are correlated [18]. In our study, the level of FSH remained between the reference values for the majority of patients (peripheral level of FSH at the early follicular phase of the menstrual cycle was 8.73 ± 4.69 IU/L). Patients with anti-FSH IgM and FSH correlation had theirs' hormonal level rather lower than in other patients and their infertility was not caused by immune system dysregulation [73]. These results suggest anti-FSH antibodies being primarily the naturally occurring antibodies rather than markers for autoimmunity against FSH hormone. This hypothesis is further supported by the discussion provided by Thomas [96] who concluded that physiological hormone levels remain below a critical threshold for the stimulation of relevant autoimmune reactions [96]. The reason for the correlation between anti-FSH IgM and the level of peripheral hormone is still unknown but could be associated with regulation of FSH bioactivity or with cyclic changes in ovary. The ovulatory process has been compared to a classical local inflammatory reaction and leukocytes have been suggested to participate actively in the cyclic events in the ovary [97–99]. Recently, cumulus and granulosa cells were shown to express cell surface signaling molecules known as pattern recognition receptors acting as sensors of the external environment important for the innate immune system to discriminate self from nonself or altered self antigens [100]. Moreover, a distinct group of mature B-lineage cells, termed B-1 cells are believed to produce IgM natural antibodies, which interact with variety of self determinants and may also cross-react with bacterial antigens [101]. The natural IgMs represent a primitive innate-like layer of adaptive immune system to provide a primary line of defence against systemic infection from viral and bacterial pathogens. There is also evidence that the natural antibodies may contribute to the elimination of autoantigens exposed during tissue damage, for instance, [101].

In addition to the presence in female serum and in ovarian tissue, FSH is also introduced to the genital tract mucosa as a constituent of semen [102]. Female immune system recognizes and reacts to the constituents of semen during insemination, a phenomenon called seminal “priming”. Its appropriate activation to induce sperm-prone mucosal tolerance facilitates subsequent pregnancy by sustaining “semiallograft” embryo development [103, 104]. During the process of partner-specific tolerance, cell-mediated and humoral immune reactions are initiated along with the production of antibodies against semen-specific and shared maternal antigens [103], such as FSH [102]. Therefore, the anti-FSH IgA antibodies detected in the female circulation could be alloantibodies initiated by semen. According to this hypothesis, levels of anti-FSH IgA would be, depending on how closely tolerance is induced, correlated with IgA antibodies produced against sperm surface antigens. We studied the correlations among patients with regard to their similarities in immunotolerating conditions in the genital tract: (i) tubal factor infertility group—women with tubal factor infertility and normal semen quality observed in their partners, (ii) male factor infertility group—healthy women and impaired sperm quality observed in their partners, and (iii) combined group of patients—women with endometriosis, PCOS or unexplained infertility and normal semen quality observed in their partners [74]. Among all subtypes of antibodies, anti-FSH IgA and anti-sperm IgA were in correlation in combined group of patients [74]. These results suggest that both detected antibodies share the antigenic origin and we propose anti-FSH IgA represent a natural activation of female immune system in inducing the mucosal tolerance to partner antigens. This idea is supported by the previous study, where anti-FSH-*β*-chain antibodies were shown to be absent in the sera of children [71].

Somewhat surprisingly, this correlation was only seen in IVF patients with PCOS, endometriosis, and unexplained infertility and not in patients with male factor or tubal factor infertility [74]. The common feature for the former three infertility groups is disturbed regulation of the immune system [19, 24, 25, 57, 58]. Disruptions of the immune system perturb the female's immune response to semen that is necessary for partner-specific tolerance and thereafter elimination of activated clones to prevent autoimmunity during pregnancy [103]. Semen exerts its “tolerance inducing” effect due to immunomodulating factors, most importantly transforming growth factor *β*_1 _(TGF*β*_1_) [105, 106]. Seminal levels of TGF*β*_1_ correlate with sperm concentration in ejaculate [105], the most decisive criterion for diagnosing male infertility. However, there is some evidence that male factor infertility is not associated with altered TGF*β*_1_ levels [107]. Although we did not distinguished subgroups of patients with male infertility by sperm parameters, generally their levels of antisperm and anti-FSH antibodies, or correlations between the two, were similar to other patients. Unlike other IVF patients participating in our study, patients with infertility caused by tubal factor do not have disturbances in female immune system regulation or seminal environment. Thus, the diagnosis-restricted correlation of antisperm and anti-FSH IgA cannot be easily explained. However, higher levels of anti-FSH IgA showed an association with the presence of the *HLA-DQB1∗03* allele [73]. In this context, it is interesting to refer to the published associations between the *HLA-DQB1∗03* allele, and the presence of the sperm-immobilizing antibodies in cervical secretions [108]. Higher production of antisperm antibodies has been detected in patients with increased intestinal permeability in bowel inflammatory disease, as a result of immunization against intestinal microbes, which seems to share common antigenic epitopes with spermatozoa [109]. Consequently, the elevated levels of anti-FSH IgA antibodies in IVF patients could be explained by an upregulation of the normal mucosal immune response. Another possible explanation of the increased anti-FSH IgA in IVF patients could be a deficits in producing antibodies that neutralize anti-FSH immunoglobulins, which has been noted in patients who produce antisperm antibodies [110]. These results together suggest that the elevated values of anti-FSH IgA in IVF patients could represent a failure in mucosal tolerance in the genital tract, which could be genetically determined. 

The production of anti-FSH IgG and IgM is decreased during uncomplicated pregnancy [75]. This decrease cannot be easily explained by the general view of a shift towards Th2 cytokines favouring humoral immunity during pregnancy [111]. However, in fact, actual elevations of autoantibodies have been detected in patients with pregnancy loss or recurrent abortion rather than in healthy noncomplicated pregnancy [111, 112]. Therefore, we believe that the development of the FSH-antibodies could reflect some other pregnancy-associated mechanism and that anti-FSH antibodies could be the natural antibodies also in this occasion. 

**Figure 1:**
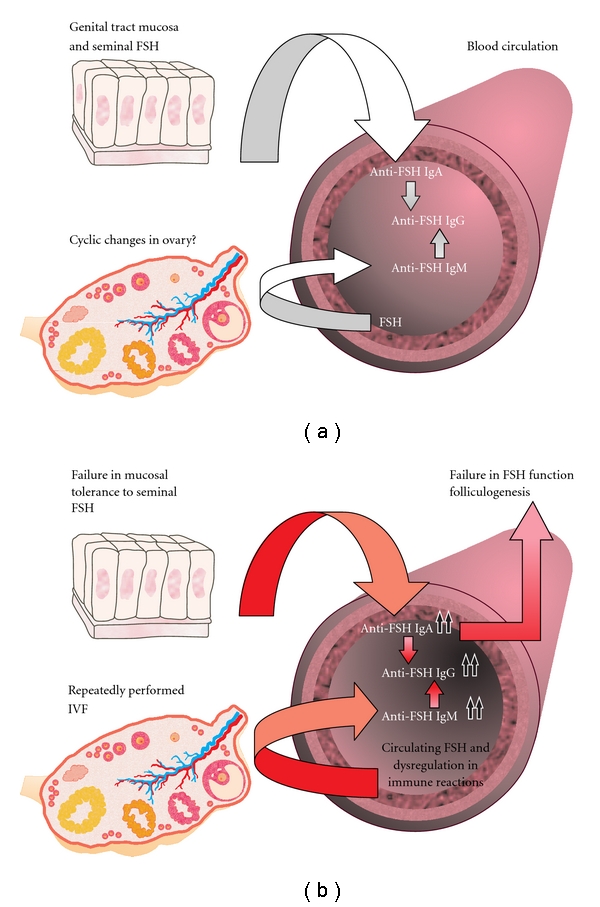
(a) Schematic overview of anti-FSH antibodies in healthy female. Antibodies detected against FSH could be natural antibodies also subjected to pregnancy-associated immune system regulations. Anti-FSH IgA detected in female circulation could be a part of the mucosal response involved in inducing immune tolerance to seminal constituents. Anti-FSH IgM associates with the peripheral level of FSH hormone and possibly contributes along with the mucosal-associated induction of IgA to the production of circulating anti-FSH IgG. (b) Increased production of naturally occurring anti-FSH antibodies in case of female infertility. The production of anti-FSH IgM and IgG antibodies could be related to a general propensity to autoimmunity or to previous IVF treatments. The elevated values of anti-FSH IgA could be explained by a genetically determined failure in mucosal tolerance in the genital tract. Anti-FSH IgG and IgA antibodies, present in sera, accumulate into the preovulatory follicle, where they affect negatively oocyte maturation.

Figure 1(a) summarizes anti-FSH as natural antibodies in healthy women. Humoral immune memory associated with natural antibody-producing B-cells might contribute to the homeostasis of the internal milieu. These cells are also believed to be responsible for autoantigen-mediated clonal selection in the process of initiating autoimmune reactions [101].

## 6. Increased Production of Anti-FSH Antibodies Contributes to Female Infertility

### 6.1. Higher Values of Anti-FSH in Infertile Women

We observed that anti-FSH antibodies were predominantly produced in infertile patients compared to healthy female blood donors [73, 75]. As stated earlier, a group of infertile patients from our studies were indicated for IVF, but serum samples were obtained before the administration of exogenous FSH [73]. Thirty-four percent of patients had had at least one previous IVF procedure, but at least three months had passed since the last FSH controlled ovarian hyperstimulation (COH). Furthermore, using stratification by previous IVF procedures, anti-FSH antibody levels were also increased in IVF patients who had never undergone IVF procedures before. The further analysis demonstrated no significant differences in anti-FSH antibody levels between the combined groups of patients with tubal and male factor infertility compared to the women with PCOS, endometriosis, unexplained infertility, and female infertility due to the other causes [73]. These data together suggest that infertility itself, rather than the cause of infertility, could be a predictive factor for the emergence of anti-FSH antibodies, as previously concluded in case of AOA [113]. The intriguing question of what associates the production of anti-FSH antibodies and female infertility stemmed directly from this context.

Female infertility has been shown to be associated with a higher occurrence of autoantibodies [17, 19, 23–25]. Except disease-specific autoantibodies described in case of endometriosis and POF [22, 48, 49], autoantibodies detected in infertile patients [17, 19, 23–25] are usually not specific to infertility or to the gynaecological diseases leading to infertility. Thus, a general immune dysbalance and activation of autoimmune processes are expected to be characteristic for female infertility [12]. We have assessed a potential susceptibility of a patient to autoimmunity by the presence of at least 1 out of 7 common IgG type of autoantibodies in relation to the autoimmunity-prone *HLA-DQB1* alleles [73]. Anti-FSH IgM associated with the production of common autoantibodies and this association was not confounded by the presence of *HLA-DQB1* alleles [73]. Our results along the ones from the literature discussed above indicate that the increased production of anti-FSH IgM could be related to a general propensity to autoimmunity in infertile women. 

The female infertility has often been studied in the context of IVF. The follicular puncture performed in IVF, in particular, can induce the production of AOA [17]. In concordance with these data, we showed that the level of anti-FSH IgM was higher in the patients who had undergone previous IVF procedures [73]. The association was revealed among IVF patients who were suffering from PCOS, endometriosis, unexplained infertility, and infertility due to the other causes but not among the women with tubal or male factor infertility. These results encourage us to speculate that repeatedly performed ovarian punctures do not enhance antiovarian autoimmunity unless a patient's infertility is caused by the diseases associated with disturbances in immune regulation [17, 19, 23–25]. However, simply based on the association study performed by us, we cannot substantiate whether the antibodies themselves may cause the need for multiple IVF procedures, or alternatively, the use of IVF procedure *per se *may enhance the production of anti-FSH. 

The receptor-binding and hormone specificity determining *β*-subunit of FSH hormone is coded by *FSHB *gene at the 11p13 [86]. Similarly to insulin gene polymorphisms affecting central tolerance through the level of gene expression in thymus [16], we were looking for an association between the two *FSHB* core haplotypes [95] and autoimmunity against FSH. As we could not detect such relationship [73], we suggest that either these single nucleotide polymorphisms do not affect gene expression in the thymus during central tolerance induction or that *FSHB*-associated autoimmunity to FSH depends on *HLA-DQB1* allelic variants other than those evaluated in our study [73]. 

The production of anti-FSH IgA is probably related to different factors than those involved in the production of anti-FSH IgM [73]. Anti-FSH IgA were associated with the presence of the *HLA-DQB1∗03* allele [74] but not with the cause of infertility, the history of previous IVF attempts or the presence of other autoantibodies [73]. Therefore, it would be tempting to speculate that anti-FSH IgA could not be autoantibodies but alloantibodies triggered by seminal FSH [102] and originating from mucosal response, as discussed above. The reasons for an increased production of this particular IgA isotype of antibodies in IVF patients, however, remain unclear. 

Correlation analysis of anti-FSH antibody values among healthy controls showed that the levels of anti-FSH IgM and IgA correlated both with the values of anti-FSH IgG [73]. There is some indirect evidence that anti-FSH IgG antibodies may, however, further worsen female fecundity by reducing the FSH functionality [70, 72]. These data lead us to investigate the effect of anti-FSH antibodies on folliculogenesis and developing infertility in women.

### 6.2. Effect of Serum Anti-FSH on Folliculogenesis

IVF has become a promising treatment for various causes of infertility. However, the success of attaining pregnancy following IVF depends on the effectiveness of COH. Serum levels of anti-FSH IgG and IgA, but not IgM antibodies at the day of oocyte retrieval, were in linear association with poorer outcome of COH [114]. The outcome of COH was defined by the duration of FSH stimulation or the total FSH required attaining an adequate response, the number of follicles punctured or oocytes obtained after COH, the number of mature oocytes or embryos, and the amount of FSH required per all of these parameters. The role of anti-FSH antibodies revealed in our study was quite remarkable. For example, our data suggest that a unit of difference in anti-FSH IgG was associated with a 220.6 IU increase in FSH needed for one zygote, while the mean amount of FSH per zygote was only 443.8 ± 401.2 IU. Furthermore, the cutoff value of >1.0 for anti-FSH IgA and IgG was calculated to be implicated to poor ovarian response (≤3 oocytes) [114]. Series of dilutions of mouse anti-human-FSH monoclonal IgG antibody were used in ELISA test to create a concentration curve and to predict serum anti-FSH IgG antibody concentration. According to the curve, the levels of anti-FSH IgG > 1.0 was presumed to correspond to the antibody levels higher than 0.5–0.6 mg/L and could, therefore, represent 0.004% of expected amount of total IgG (8–17 g/L) in peripheral blood. The same or even slightly lower levels of blocking and stimulating serum TSH-receptor autoantibodies has been demonstrated previously in patients with Graves' disease and in autoimmune hypothyroidism [115]. Since anti-FSH antibodies are often detected in patients with AOA [71, 116] our results may simply reflect an impaired ovarian function due to ovarian autoimmunity. The association between antigonadotrophin [72] or AOA [67] IgG in the sera at oocyte retrieval and poor ovarian response to the FSH stimulation has been shown previously. 

In addition to reflecting ovarian autoimmunity, anti-FSH antibodies may impair the function of exogenous or endogenous FSH. For example, anti-FSH could form immune complexes with FSH and induce its clearance, as recently shown for creatine kinase in patients with corresponding antibodies [117]. Also, anti-FSH could interrupt the binding of FSH to its receptor. This hypothesis is supported by our data suggesting anti-FSH antibodies in sera correlated with antibodies directed against the 78–93 amino acid region of the *β*-chain of the human FSH [71, 75], the domain that determines FSH receptor binding specificity [86]. On the other hand, the study of *in vitro* FSH-blocking ability of anti-FSH IgG in women with good IVF response [70] suggested that anti-FSH antibodies may be nonpathogenic. However, this study did not specify which FSH epitopes were bound by the pool of anti-FSH antibodies.

Although the pathophysiology of anti-FSH in association with poor ovarian response is still unclear, the importance of these antibodies is noteworthy. Woman's age and her ovarian volume and the number of follicles counted at the early follicular phase of her spontaneous menstrual cycle were significant clinical parameters predicting the outcome of COH [114], as also demonstrated by others [118]. Yet, anti-FSH antibodies could represent an additional importance to the clinical parameters like age, follicle number, or ovarian volume in predicting the outcome of COH. Furthermore, if the influence of anti-FSH on the ovarian response is revealed in the IVF patients (where supraphysiological amounts of FSH were administered to stimulate folliculogenesis), the importance of those antibodies in unstimulated spontaneous folliculogenesis might be substantial.

### 6.3. Changes in Serum Levels of Anti-FSH during COH in Relation to Follicular Fluid

Serum levels of anti-FSH IgG and IgA, but not IgM antibodies, decreased following COH, conducted with GnRH antagonist protocol [114]. Although interpretation of these results is not straightforward, we believe the decrease in anti-FSH antibody levels could partly be explained by the supraphysiological levels of immunosuppressive progesterone and testosterone [114, 119, 120] produced in COH. This hypothesis is supported by our previous data suggesting an overall decrease in the number of common IgG autoantibodies during COH [57]. Additionally, anti-FSH antibodies could form immune complexes with administered recombinant FSH or with endogenous FSH (produced in pituitary prior to administration of GnRH antagonists), resulting in the decrease in antibody levels. However, the levels of anti-FSH IgM remain unchanged after COH [114]. As IgM antibodies also form immune complexes, the reactivation of the immune system towards novel epitopes on the FSH molecule and the production of anti-FSH IgM during COH might be speculated. As well, immunization against exogenous gonadotrophins has also been previously suggested [72]. This hypothesis is further supported by our findings and that found from the literature that an increase in IgM type of anti-FSH [73] and AOA [17, 32, 73, 121] associated with repeated IVF procedures. However, it was also reported that AOA were initiated by ovarian puncture rather than administered FSH [17]. Additionally, circulating anti-FSH could pass into the follicular fluid during follicle maturation; however, this decrease would hardly be detectable in sera by current laboratory tests. 

The charge- and size-selective ovarian blood-follicle barrier is open for IgG to pass into the follicular fluid [122] and the concentration of total IgG and IgA in follicular fluid as well as in blood should be equivalent [123]. We have measured the presence of anti-FSH IgG, IgA and IgM in negligible amounts in follicular fluid [114]. The level of anti-FSH IgA also correlated with the level of same antibody in peripheral blood [114]. However, anti-FSH IgG seemed to accumulate into the growing follicle, since the concentration of follicular anti-FSH IgG associated positively with the diameter of a follicle, reflecting the maturity of a follicle [114]. The increase in follicular anti-FSH IgG with the growth of the follicle is not a simple reflection of anti-FSH IgG serum levels, as serum anti-FSH IgG levels significantly decreased during COH [114]. Logically, follicular anti-FSH IgG levels correlated with the amounts of recombinant FSH used for COH and FSH levels measured in the follicle [114]. The level of follicular FSH increases while the follicle grows [124, 125], and expectedly, follicular FSH correlates with the amount of FSH administered exogenously [67, 114]. Thus, anti-FSH IgG could diffuse along with the antigen to the follicular fluid during the COH. Although anti-FSH IgA and IgM were detected in the follicle, levels of these antibodies were not associated with follicle diameter [114], which is in agreement with other authors [126]. In addition, anti-FSH IgM levels in the follicle were very low compared to serum antibody levels [114], in concordance with that reported by Clarke and coworkers [123], where total IgM in the follicle represented approximately 10% of its plasma concentration [123].  Figure 1(b) summarizes our studies on anti-FSH antibodies in cases of female infertility. These results emphasize the need for further research to elucidate the clinical relevance of anti-FSH antibodies in the spontaneous menstrual cycles.

Finally, low-dose prednisolone therapy has improved pregnancy rate in patients with recurrent IVF failure [62, 67, 127] and in non-IVF patients [128]. Different treatment regimes of oral prednisolone has been suggested, such as 10 mg/d during one month prior to the COH [62], 0.5 mg/kg/d starting from the beginning of COH until the end of 1st trimester of pregnancy, and followed by lowering the dose thereafter [127], or 10 mg/d in the 1st week, 5 mg/d in the 2nd week, 2.5 mg/d in the 3rd week, and 2.5 mg/d 3 times a week during the last (4th) week before intrauterine insemination [128]. However, considering the time duration of ovarian folliculogenesis, the treatment should start at least 1-2 months before COH [67]. Most benefit of immunosuppressive treatment can gain infertile patients who represent antiovarian autoimmunity [129]. Testing serum anti-FSH antibodies could help infertility treatment specialists to identify those patients.

## 7. Conclusions

Female fertility can be affected by diseases or dysfunctions of reproductive tract, neuroendocrine system, and immune system. Reproductive autoimmune failure can be associated with overall activation of immune system or with immune system reactions specifically directed against ovarian antigens. Antiovarian autoantibodies are mostly directed against *β*-subunit of follicle stimulating hormone (anti-FSH). Anti-FSH could be natural antibodies. Anti-FSH IgA detected in female circulation could be a part of the mucosal response involved in inducing immunotolerance to seminal constituents. Anti-FSH IgM associates with the peripheral level of FSH hormone and contributes along with the mucosal-associated induction of IgA to the production of circulating anti-FSH IgG. Additionally, higher production of anti-FSH antibodies could contribute to female infertility. The induced production of anti-FSH IgM antibodies could be related to a general propensity to autoimmunity or to previous IVF treatments. The elevated values of anti-FSH IgA could indicate genetically determined failure in mucosal tolerance in the genital tract. Serum IgG and IgA anti-FSH antibodies, measured at the day of oocyte retrieval, predict the outcome of ovarian stimulation, additionally to that observed with age and other clinical parameters characterizing the ovarian reserve. A population of anti-FSH antibodies which are produced against 78–93 epitope on the *β*-chain might modulate the recognition and binding of FSH to its receptor and might, therefore, have a pathological influence on ovarian function. We have also demonstrated that anti-FSH IgG, IgA, and traces of IgM antibodies were detectable in the follicular fluid and that anti-FSH IgG antibodies accumulated into the preovulatory follicle. Immunosuppressive treatment could improve the pregnancy rate in anti-FSH seropositive infertile patients.

## Figures and Tables

**Table 1:** Etiology of female infertility (based on the diagnostic and treatment guidelines provided by [1, 2]).

*Anovulatory infertility*
Hyperprolactinaemia
Pituitary adenoma
Hypogonadotrophic hypogonadism
Kallmann's syndrome
Weight loss
Hypergonadotropic hypogonadism
Premature ovarian failure (POF) and early menopause
Gonadotrophin resistance due to a receptor defect
“Normogonadotropic” oligoanovulation
Polycystic ovary syndrome (PCOS)
Adrenal cause of hyperandrogenism
Genetic determinants
Turner syndrome, Swyer syndrome
Androgen insensitivity syndrome
Androgen synthesis disorders

*Tuboperitoneal infertility*

Tubal factor infertility
Endometriosis

*Autoimmunity*

POF
Recurrent pregnancy loss
Autoimmunity-associated infertility

*Uterine abnormalities*

Malformations
Submucous myomas
Endometrial adhesions

*Unexplained infertility*

**Table 2:** Serum autoantibodies in female infertility and infertility-related diseases.

Patients (N)	Autoantibodies	Methods	Study design	Authors (reference no.)
POF				

POF (45)	AOA 47%* Antioocyte Aab 47%* AOA or anti-oocyte Aab 69%* Anti-LH 6.7% (also AOA positive) AThA 18%* Antiplacental Aab 22%*	ELISA	CC	Luborsky et al. 1990 [30]

POF (45)	AOA 24–60%*	ELISA	CC	Wheatcroft et al. 1994 [68]

POF (48)	Anti-3 beta hydroxysteroid dehydrogenase Aab 21%*	IB, IF, cDNA screening	CC	Arif et al. 1996 [69]

POF (46)	AOA IgG, IgA or IgM 59%—IgG 74.1%, IgA 33.3%, IgM 29.6%*	ELISA	CC	Fénichel et al. 1997 [29]

(A) POF (14) (B) IVF poor responders (29) (C) IVF good responders (14)	FSH blocking IgG: (A) 21.4% (B) 6.9% (C) 85%*	IgG purification, cell culture exposure	CC	Reznik et al. 1998 [70]

POF (30) Unexplained infertility (38)	AOA and AThA 60%* ANA and ACA 16%* AOA 53%* AThA 30%*	EIA	CC	Luborsky et al. 1999 [32]

POS positive for AOA (36)	Anti-FSH (anti-V14D) 94.4%*	ELISA, IB, IF, peptide screening	P	Gobert et al. 2001 [71]

POF (15)	AOA 66.6%* Antizona pellucida Aab 53.3%* TMA 33.3%	IHC	CC	Kelkar et al. 2005 [31]

IVF patients				

IVF poor responders with male infertility or TFI (26)	AOA 77%* Anti-FSH 92%* Anti-LH 65%*	ELISA	CC	Meyer et al. 1990 [72]

IVF failure (80)	AOA 12.5%*	IF	CC	Geva et al. 1999 [62]

IVF failure (17)	1 out of 6 common Aab IgG 82.3%*: ACA 58.8% LA 47.1% AThA 58.8% ANA 58.8% SMA 11.8%	ELISA, IF, PDCA	CC	Putowski et al. 2004 [61]

IVF patients (135): (A) PCOS, endometriosis, unexplained infertility (B) TFI or male infertility	(A) and (B) higher titer of anti-FSH IgG, IgA and IgM* (A) 1 out of 7 common Aab IgG 49%*-ANA 2 preparations, SMA, PCA, ACA, B2-GPI or anti-TPO	ELISA, IF	CC	Haller et al. 2007 [73]

IVF poor responders (16)	Anti-FSH IgA 37.5%* Anti-FSH IgG 31.3%*	ELISA	CC	Haller et al. 2008 [74]

TFI with IVF failure (156)	AEA IgA* (antialpha enolase)	IB, MS	CC	Sarapik et al. 2010 [43]

TFI (21)	Antichlamydial HSP60 antibody titer*	ELISA, IB, IF	CC	Rodgers et al. 2010 [42]

Non-IVF infertility patients				

(A) Unexplained infertility (26) (B) Unexplained abortion (24)	2 APA, 5 antihistone or 4 antipolynucleotide IgG, IgA or IgM (A 88% and B 70.8%)	ELISA	P	Gleicher et al. 1989 [66]

Pregnancy complications (69): (A) Early pregnancy loss (B) Foetal death (C) Preeclampsia	AThA 37.7%*: (A) 37.9%* (B) 40.9%* (C) 33.3%*	ELISA, PDCA, RIA	CC	Mecacci et al. 2000 [11]
Infertility (108): Menstrual cycle disturbances Anovulation Luteal phase deficiency Unexplained infertility PCOS Endometriosis	1 out of 9 common Aab IgG 40.7%* ANA 13.9%* SMA 27.8%* TMA 1.9%* PCA 0.6% B2-GPI 4.4% ACA 5%	ELISA, IF	CP	Reimand et al. 2001 [25]

Infertility (438): Endometriosis TFI Ovarian dysfunction Male infertility Unexplained infertility	Anti-TPO 14%: 18% in female infertility* 29% in endometriosis*	RIA	CC	Poppe and Velkeniers 2002 [60]

(A) Infertility (178)—PCOS, endometriosis (B) Uncomplicated pregnancy (75)	(A) higher titer of anti-FSH (anti-V14D) IgA* (B) lower titer of anti-FSH (anti-V14D) IgG, IgM*	ELISA	CC	Haller et al. 2005 [75]

Infertility-related diseases				

Endometriosis (13)	AOA, AEA, anti-theca cell Aab, anti-granulosa cell Aab titers*	IF, PHA	CC	Mathur et al. 1982 [50]

Endometriosis (59)	ANA 28.8%, LA 45.5% (inversely related to disease stage) 1 out of 16 antigens IgG 64.5% 1 out of 16 antigens IgM 45.2%	IF, PDCA	P	Gleicher et al. 1987 [21]

Endometriosis (60)	Anti-*α* 2HS glycoprotein and antitransferrin titers*	ELISA	CC	Mathur et al. 1999 [49]

PCOS (34)	AOA IgG, IgA or IgM 44%—IgG 27%, IgA 3%, IgM 27%*	ELISA	CC	Fénichel et al. 1999 [19]

*Statistically significant compared to the reference (*P* < 0.05), Aab-autoantibodies, ACA-anticardiolipin autoantibodies, AEA-antiendometrial autoantibodies, ANA-antinuclear autoantibodies, AOA-antiovary autoantibodies, APA-antiphospholipid autoantibodies, AThA-anti-thyroid autoantibodies, B2-GPI-anti-beta 2-glycoprotein I autoantibodies, EIA-enzyme immunoassay, ELISA-enzyme-linked immunosorbent assay, FSH-follicle stimulating hormone, HSP-heat shock protein, IF-immunofluorescence, IB-immunoblot analysis, IHC-immunohistochemistry, IVF in vitro fertilization, LA-lupus anticoagulant, CC-case-control study, CP-cases-population study, LH-luteinizing hormone, MS-mass spectrometry, P-prevalence, PCA-parietal cell autoantibodies, PCOS-polycystic ovary syndrome, PDCA-phospholipid-dependent clotting assay, PHA-passive haemagglutination, POF-premature ovarian failure, RIA-radioimmune assay, SMA-smooth muscle autoantibodies, TFI-tubal factor infertility, TMA-thyroid microsomal autoantibodies, TPO-thyroid peroxidase, V14D-78-93 amino acid immunodominant epitope on FSH.
